# Development of a Blocking ELISA for the Serological Diagnosis of Getah Virus

**DOI:** 10.1155/tbed/6257918

**Published:** 2026-06-23

**Authors:** Shiyi Shen, Zhengxin Yang, Yumei Sun, Weidong Yan, Mengjia Zhang, Hailong Ma, Wenjuan Du, Ahmed H. Ghonaim, Anan Jongkaewwattana, Suphot Wattanaphansak, Qigai He, Wentao Li

**Affiliations:** ^1^ National Key Laboratory of Agricultural Microbiology and Hubei Hongshan Laboratory, Huazhong Agricultural University, Wuhan, 430070, China, hzau.edu.cn; ^2^ The Cooperative Innovation Center for Sustainable Pig Production, Huazhong Agricultural University, Wuhan, 430070, China, hzau.edu.cn; ^3^ College of Veterinary Medicine, Huazhong Agricultural University, Wuhan, 430070, China, hzau.edu.cn; ^4^ Hainan Research Institute, Huazhong Agricultural University, Sanya, China, hzau.edu.cn; ^5^ Wuhan Keqian Biology Co. Ltd., Wuhan, 430072, China; ^6^ College of Veterinary Medicine, Henan Agricultural University, Zhengzhou, 450046, China, henau.edu.cn; ^7^ National Center for Genetic Engineering and Biotechnology, Pathum Thani, 12120, Thailand, biotec.or.th; ^8^ Department of Veterinary Medicine, Faculty of Veterinary Science, Chulalongkorn University, Pathum Wan, 10330, Bangkok, Thailand, chula.ac.th; ^9^ Hubei Jiangxia Laboratory, Wuhan, 430200, China

**Keywords:** blocking ELISA, diagnosis, E2 protein, getah virus, monoclonal antibody

## Abstract

Getah virus (GETV) is an emerging arbovirus that poses a growing threat to the swine industry because of its ability to infect pigs and other animals. However, the lack of an effective porcine GETV vaccine and commercially available diagnostic tools has hindered control efforts. In the study, we expressed the recombinant GETV E2 protein in a mammalian cell system to preserve its native conformation and antigenicity and generated monoclonal antibodies (mAbs) by immunizing mice with this protein. Using the GETV E2 protein and horseradish peroxidase (HRP)–labeled mAbs, we successfully established a rapid and specific blocking ELISA for the serological detection of GETV. The assay was comprehensively evaluated for specificity, reproducibility, and consistency with the reference immunofluorescence assay (IFA), demonstrating reliable diagnostic performance. In a clinical evaluation involving 462 samples, the assay showed a positive rate of 12.121%, confirming its practical applicability. This novel blocking ELISA provides a valuable tool for vaccine development and epidemiological investigations, contributing to more effective strategies for GETV control.

## 1. Introduction

Getah virus (GETV) is a single‐stranded, positive‐sense RNA virus belonging to the family *Togaviridae* and the genus *Alphavirus*. It is a classic arbovirus, primarily transmitted by mosquitoes. GETV was first isolated from *Culex* spp. in Malaysia in 1955 and designated strain MM2021. For decades, isolates were predominantly isolated from mosquitoes until 1978, when strain MI‐110 was isolated from horses during an outbreak in Ibaraki, Japan, marking the first identification of GETV in mammals. Since then, GETV has been increasingly reported in a wide range of hosts, including pigs [1], pangolins [2], bats, red pandas (*Ailurus fulgens*) [3], red‐bellied tree squirrels, and foxes [4]. The virus is now spreading across Asia [5–7], Europe, and Oceania [8], posing a growing threat to animal health and the livestock industry.

**Figure 1:**
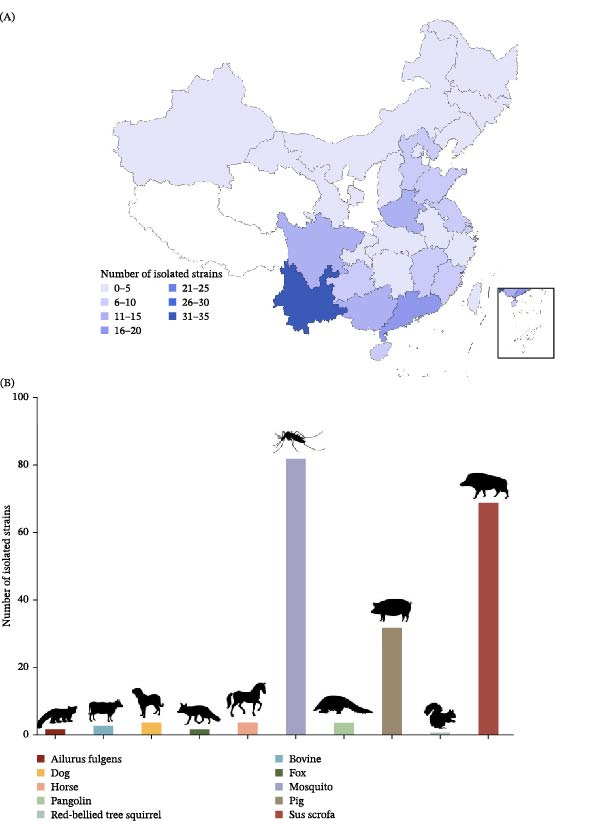
Isolation of the GETV strains in China. (A) Isolation of GETV in different provinces of China. (B) Isolation of GETV in different hosts.

According to publicly available sequence records retrieved from the National Center for Biotechnology Information (NCBI) using the keyword “GETV,” researchers have mapped GETV isolates across various Chinese provinces and animal hosts (Figure 1), based on data available up to November 24, 2024. These findings reflect the increasing geographic and host range of GETV in China, underscoring its emergence as a spreading zoonotic virus in the region.

One of the earliest documented animal outbreaks occurred in 1985 in Kanagawa, Japan, where infected piglets exhibited symptoms including depression, tremor, diarrhea, and death [9]. Subsequent studies confirmed that GETV infection can cause fever, anorexia, reproductive disorders, and vertical transmission [10, 11]. A severe outbreak occurred in 2017 in Hunan Province, China, leading to the deaths of over 200 piglets and causing reproductive failure in more than 150 sows, severely affecting local swine production. Histopathological examinations revealed severe tissue damage in infected piglets, confirming the pathogenic nature of the virus [1]. Experimental studies in mice have demonstrated that GETV can cross the placental barrier and impair male reproductive function by reducing semen quality [12], further emphasizing its impact on fertility and reproduction [13]. Currently, no specific antiviral treatment exists for GETV in animals. However, doxycycline has shown potential antiviral effects by inhibiting the replication of GETV [14]. Although an equine vaccine based on the 1978 GETV strain was developed, it failed to provide adequate protection [15]. More recently, a virus‐like particle (VLP) vaccine demonstrated protective effects in mice against viremia and arthritis [16], offering promising potential for future use in pigs and horses.

In the absence of effective vaccines or therapeutic options, accurate and reliable diagnostic methods are critical for controlling GETV. Recent advances include molecular assays, such as an RT‐LAMP for field detection, and serological methods, including indirect ELISA [17, 18]. These tools have been used to monitor GETV prevalence, with positivity rates of 37.59% in some regions of China. GETV encodes five structural proteins: capsid, E3, E2, 6K, and E1. Among these, the E2 glycoprotein plays a pivotal role in viral attachment to host cells, facilitating infection [19]. It is also highly conserved among Chinese strains (20 and 22), making it an ideal target for both diagnostic and vaccine development.

**Figure 2:**
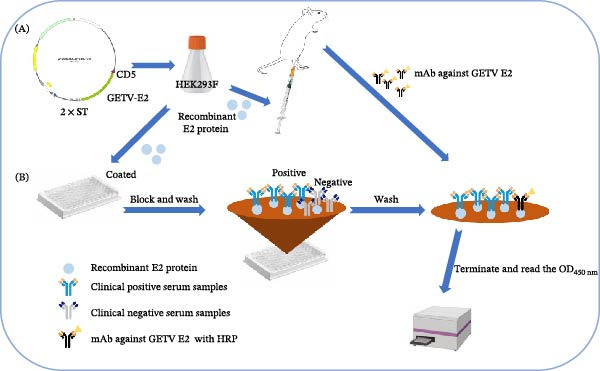
Schematic flowchart of blocking ELISA development. (A) The process of the eukaryotic expression of the recombinant E2 protein. (B) The process of the development of the blocking ELISA.

To address the need for a sensitive serological tool, we developed a blocking ELISA based on the E2 glycoprotein (Figure 2). We successfully expressed recombinant E2 protein using the HEK‐293F expression system and generated monoclonal antibodies (mAbs) from E2‐immunized mouse hybridomas. These E2‐specific mAbs enabled the development of a highly specific and effective blocking ELISA, providing a powerful tool for sero‐surveillance, vaccine evaluation, and epidemiological investigations.

## 2. Materials and Methods

### 2.1. Virus and Serum Samples

The 4–8‐week‐old, female, BALB/c mice were purchased from the Laboratory Animal Center of Huazhong University. The animal experiment was in strict accordance with the Guidance for the Care and Use of Laboratory Animals of Huazhong University (SYXK(HB)‐2020‐0019). The GETV‐YL strain (GenBank accession OL352731) was isolated previously and preserved in our laboratory [20]. The GETV‐positive and GETV‐negative pig serum samples were identified by immunofluorescence assay (IFA) and preserved for assay development. The PCV2, PRV, PRRSV, and CSFV‐positive serum was preserved in our laboratory. Four hundred sixty‐two pig serum samples were collected from the Animal Disease Diagnostic Center, Huazhong Agricultural University.

### 2.2. Construction of Recombinant E2 Expression Plasmid

The E2 gene from GETV‐YL fused with Strep Tag was codon‐optimized for expression in HEK‐293F cells using the NovoPro bioinformatics tool (https://www.novopro.cn/tools/codon-optimization.html), and the optimized gene was synthesized by SynbioB Co. (Tianjin, China). Primers were designed using SnapGene 6.0.2 software. The E2 gene was amplified using primer pair E2‐F and E2‐R, and the pCAGGS vector (containing a Strep‐tag) was linearized using primer pair pCAGGS‐KL‐F/KL‐R. Both fragments were assembled by homologous recombination (ABclonal, Wuhan, China). The recombinant plasmid was verified by double digestion with PacI and EcoRI restriction enzymes, followed by sequencing.

### 2.3. Expression, Purification, and Identification of Recombinant E2 Protein

HEK‐293F cells (Thermo Fisher Scientific) were cultured in a shaker incubator at 37°C, 120 rpm, and 5% CO_2_ to a density of 1 × 10^6^ cells/mL. A PEI–DNA complex was prepared and used to transfect the cells. Following a 48‐h incubation period, the culture supernatant was harvested and purified using Strep‐Tactin resins (IBA, Göttingen, Germany). The purity and identity of the recombinant E2 protein were confirmed by SDS‒PAGE and Western blotting.

### 2.4. Production of mAbs Against GETV E2 Protein

Female BALB/c mice (4–6 weeks old) were immunized intramuscularly in the thigh with 20 μg of purified E2 protein mixed with an equal volume of QuickAntibody‐Mouse3W adjuvant (Biodragon, Suzhou, China) in a 50 μL final volume. A second immunization was administered 14 days later using the same protocol. On day 21, mice received a booster dose of E2 protein alone for three consecutive days. Three days later, splenocytes were harvested and fused with Sp2/0 myeloma cells using 50% (w/v) PEG in a 37°C water bath. Hybridomas were cultured in selective DMEM containing HATs (Sigma, St. Louis, MO, USA) for 14 days. Positive clones secreting anti‐E2 antibodies were screened by indirect ELISA and IFA and expanded in HT‐containing DMEM supplemented with 20% fetal bovine serum (FBS, ExCell Bio, Suzhou, China). Antibody subtypes were determined using an indirect ELISA kit (Biodragon, Suzhou, China).

### 2.5. Indirect ELISA

ELISA plates were coated with 1 μg/mL of purified E2 protein and incubated overnight at 4°C. After one wash with phosphate‐buffered saline with tween (PBST), wells were blocked with 5% skim milk for 2 h at 37°C. Following three washes with PBST, hybridoma supernatant was added; polyclonal antibodies (pAbs) served as a positive control, and DMEM with 20% FBS was used as a negative control. After incubation and washing, horseradish peroxidase (HRP)–conjugated goat antimouse IgG (ABclonal, Wuhan, China) was added. Plates were washed again for five washes with PBST, and TMB substrate (Solarbio, Beijing, China) was added and incubated for 10 min at room temperature. The reaction was stopped with ELISA Stop Solution (Solarbio, Beijing, China). The optical density (OD) at 450 nm was measured with a microplate reader (Omega Bio‐Tek, USA).

### 2.6. Indirect IFA

PK‐15 cells were infected with GETV‐YL at an MOI of 0.01 and incubated at 37°C and 5% CO_2_ until a 20%–30% cytopathic effect (CPE) was observed. Cells were fixed with 4% paraformaldehyde, washed with PBS, and blocked with 5% bovine serum albumin (BSA) diluted in PBS, followed by three additional washes with PBS. The mouse anti‐GETV E2 pAb served as a positive control, while DMEM containing 20% FBS served as the negative control. Alexa Fluor 488–conjugated donkey antimouse (AntGene, Wuhan, China) was used as the secondary antibody. Nuclei were stained with DAPI (Solarbio), and fluorescence was visualized using a microscope.

### 2.7. Western Blot

Purified E2 protein samples were separated by SDS‒PAGE and transferred onto PVDF membranes (Merck Millipore, Billerica, MA, USA). Membranes were blocked with 5% skim milk at room temperature and then incubated with the E2‐specific mAbs. HRP‐conjugated goat antimouse IgG (ABclonal, Wuhan, China) was used as the secondary antibody. Target protein bands were visualized using an enhanced chemiluminescence (ECL) chromogenic detection kit.

### 2.8. Development of the Blocking ELISA

To select the most suitable E2‐specific mAbs for the blocking ELISA, the titers of purified mAbs were first assessed via an indirect ELISA. The selected mAbs were then conjugated with HRP to establish the blocking ELISA for detecting GETV‐specific antibodies. Purified GETV E2 was coated onto polystyrene ELISA plates and incubated for 12 h at 4°C or 2–4 h at 37°C. Plates were washed once with PBST and blocked with 200 μL of 5% skim milk or BSA for 1–2 h at 37°C. After washing, 100 μL of diluted serum was added to each well and incubated at 37°C for different conditions. The plates were then washed, and 100 μL of HRP‐conjugated mAbs was added, followed by incubation at 37°C for 30–120 min. After further washing, 100 μL of TMB substrate solution (Solarbio, Beijing, China) was added to each well and incubated for 5–20 min at 37°C. The reaction was terminated by the addition of ELISA Stop Solution (Solarbio, Beijing, China). The OD at 450 nm was measured using a microplate reader (Omega Bio‐Tek, USA). The percent inhibition (PI) was calculated using the following formula:

PI%=OD450 value of negative controls- OD450 value of sample/OD450 value of negative controls×100%.

### 2.9. Cutoff Value, Diagnostic Sensitivity, and Specificity

Owing to the lack of commercially available GETV ELISA kits, IFA and neutralization tests were used as reference standards. The optimal cutoff value for the blocking ELISA was determined using 22 GETV‐positive and 90 GETV‐negative pig serum samples. Receiver operating characteristic (ROC) curve analysis was performed using IBM SPSS Statistics 26.0 software to assess the diagnostic sensitivity and specificity of the assay.

### 2.10. Analytical Specificity and Sensitivity of the Blocking ELISA

The analytical specificity of the blocking ELISA was assessed using a serum sample positive for ASFV, CSFV, JEV, PCV2, PEDV, PRV, and PRRSV. To determine analytical sensitivity, twofold serial dilutions of two GETV‐positive serum samples were tested by blocking ELISA and neutralization test.

### 2.11. Assessment of the Reproducibility of the Blocking ELISA

To evaluate the reproducibility of the blocking ELISA, 10 GETV‐positive and eight negative serum samples were tested. For intra‐assay repeatability, each sample was tested in triplicate on the same batch of precoated plates. For interassay repeatability, the same samples were tested using three different batches of precoated plates. The mean PI values and coefficients of variation (CVs) were calculated based on three replicates of each sample.

### 2.12. Detection of GETV Antibodies in Pig Sera

A total of 462 clinical swine serum samples collected from different regions of China between 2022 and mid‐2023 were screened using the developed blocking ELISA to detect GETV‐specific antibodies.

### 2.13. Statistical Analysis

Statistical analyses were performed using the IBM SPSS Statistics 26.0 and GraphPad Prism 8.0 software. Data were expressed as means ± standard deviations (SDs). Intra‐ and interassay variations were assessed using CVs.

## 3. Results

### 3.1. Preparation of Recombinant GETV E2 Protein

**Figure 3:**
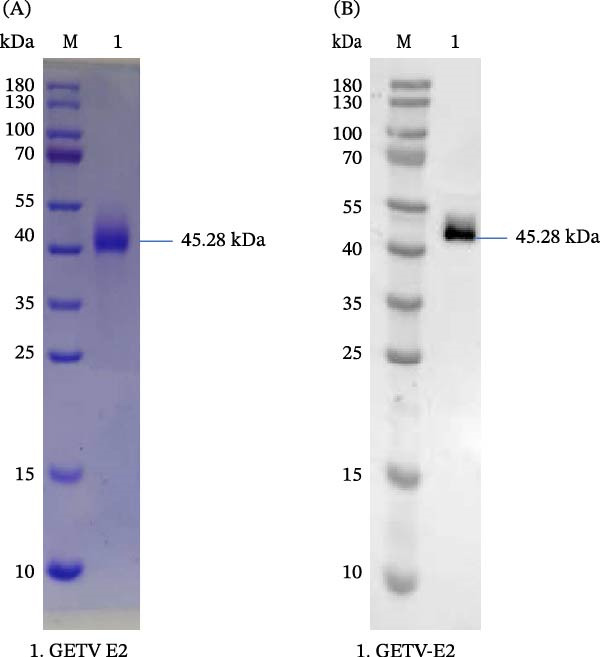
Identification of recombinant E2 protein. (A) SDS‒PAGE analysis of purified E2 protein. (B) Western blot analysis of purified E2 protein.

Recombinant GETV E2 protein was successfully expressed and purified using a Strep‐tag affinity system. SDS–PAGE analysis confirmed the expression of the target protein, revealing a band at the expected molecular weight of ~45.28 kDa, with high purity (Figure 3A). Western blot analysis further verified the identity of the protein, demonstrating specific reactivity with pAbs against GETV E2 (Figure 3B). The purified recombinant E2 protein was subsequently used as the coating antigens in the development of blocking ELISA.

### 3.2. Production and Validation of GETV E2 mAbs

**Figure 4:**
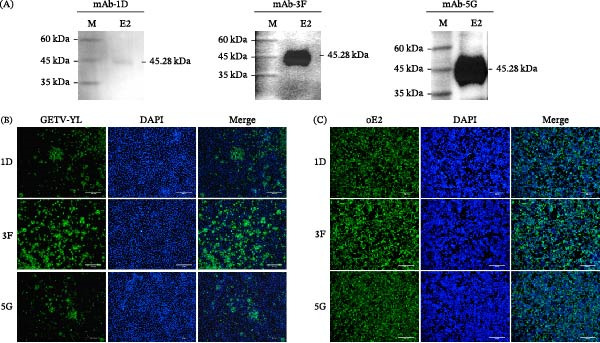
Reactivity of monoclonal antibodies (mAbs) against GETV E2 protein. (A) Western blot analysis showing the reactivity of anti‐GETV E2 mAbs with purified E2 protein (~45.28 kDa). M: molecular weight marker; lane E2: purified GETV E2 protein. (B) IFA results demonstrating the binding of the three mAbs to PK‐15 cells infected with the GETV‐YL strain. (C) IFA analysis of 293T cells transiently transfected with the GETV E2 expression plasmid. Cells were probed with E2‐specific mAbs and visualized using Alexa Fluor 488–conjugated donkey antimouse IgG (H + L).

Following two rounds of subcloning by limiting dilution, seven positive hybridoma clones, designated 1D, 2C, 3F, 5G, 10B, 11D, and 11E, were identified by indirect ELISA and IFA. Based on their growth characteristics and antibody production, clones 1D, 3F, and 5G were selected for subsequent experiments. Isotype characterization revealed that mAbs 1D and 5G belonged to the IgG1 subclass, while mAb 3F was classified as IgG2b; all three antibodies possessed kappa light chains. Western blot analysis revealed that both mAbs 3F and 5G strongly recognized the recombinant E2 protein, while mAb 1D showed no detectable reactivity (Figure 4A). IFA results demonstrated that all three mAbs (1D, 3F, and 5G) specifically reacted with the GETV‐YL strain in infected PK‐15 cells (Figure 4B). Additionally, the E2 protein expressed in transiently transfected 293T cells was specifically recognized by these three mAbs, confirming their specificity for the GETV E2 antigen (Figure 4C).

### 3.3. Selection of mAbs for Blocking ELISA Development

**Figure 5:**
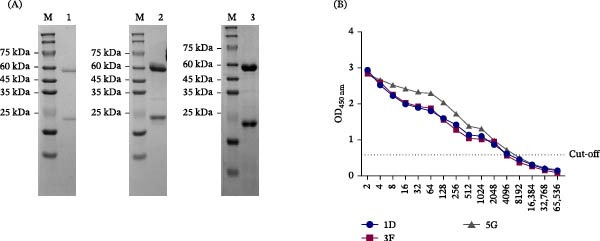
Evaluation of E2‐specific monoclonal antibodies (mAbs) for use in the blocking ELISA. (A) SDS‒PAGE analysis of the three purified mAbs. Lanes 1–3 correspond to mAbs 1D, 3F, and 5G, respectively. (B) Indirect ELISA showing the titers of the purified E2 mAbs, used to assess their suitability for blocking ELISA development.

The three candidate mAbs (1D, 3F, and 5G) were evaluated based on their blocking efficiency and antibody titers. Among them, mAb 5G demonstrated the best binding efficiency, making it the most suitable candidate for establishing the blocking ELISA (Figure 5).

### 3.4. Standardization and Determination of the Cutoff Value of the Blocking ELISA

**Figure 6:**
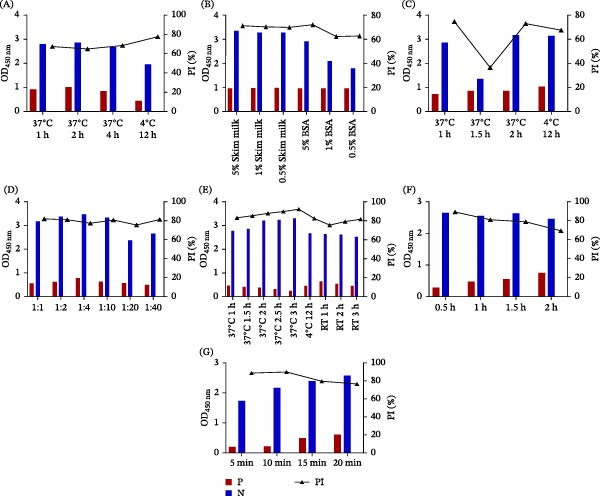
Optimization of ELISA working conditions. (A) Determination of antigen coating conditions. (B) Determination of blocking buffer. (C) Determination of the blocking condition. (D) Determination of serum dilution. (E) Determination of incubation time of samples. (F) Determination of the incubation time of HRP‐labeled antibody. (G) Determination of the reaction time of substrate.

Based on the results of checkerboard titration, optimal reaction conditions for the blocking ELISA were established. The coated antigen was used at a concentration of 0.0625 μg/mL in a volume of 100 μL per well and incubated at 4°C for 12 h (Figure 6A). Blocking was performed using 5% BSA at 37°C for 1 h (Figure 6B,C). Serum samples were diluted 1:1 with 5% BSA and incubated at 37°C for 1 h (Figure 6D,E). The HRP‐labeled mAb was diluted 1:10,000 and incubated at 37°C for 30 min (Figure 6F). Finally, the TMB substrate reaction was carried out at 37°C for 10 min (Figure 6G). These standardized conditions were used in subsequent experiments to ensure reproducibility and optimal assay performance (Table 1).

**Table 1:** Optimization of the reaction conditions for GETV blocking ELISA.

Optimized conditions	Antigen coating	Blocking conditions	Serum to be tested	HRP‐labeled antibody	TMB reaction time
Concentration/dilution	0.0625 μg/mL	5% BSA	1:1	1:10,000	—
Reaction conditions	4°C 12 h	37°C 1 h	37°C 3 h	37°C 0.5 h	37°C 10 min

After protocol optimization, a total of 22 GETV‐positive and 90 GETV‐negative serum samples were tested to determine the cutoff value of the developed blocking ELISA. ROC analysis demonstrated excellent diagnostic performance, with an area under the curve (AUC) of 1.000 (95 % confidence interval: 0.989–1.0), indicating perfect discrimination between positive and negative samples (Table 2). On the basis of the ROC analysis, samples with a PI < 40 % were considered negative, those with a PI ≥ 50% as positive, and those with PI values between 40% and 50% were considered equivocal or suspicious.

**Table 2:** ROC curve analysis results for the GETV blocking ELISA.

Area below the curve				
Area	Standard error^a^	Sig.^b^	Asymptotic to 95% confidence interval	
			Lower limit	Upper limit
1.000	0.000	0.000	1.000	1.000

^a^Under the nonparametric assumption.

^b^Null hypothesis: true area = 0.5.

### 3.5. Analytical Specificity of the Blocking ELISA

**Figure 7:**
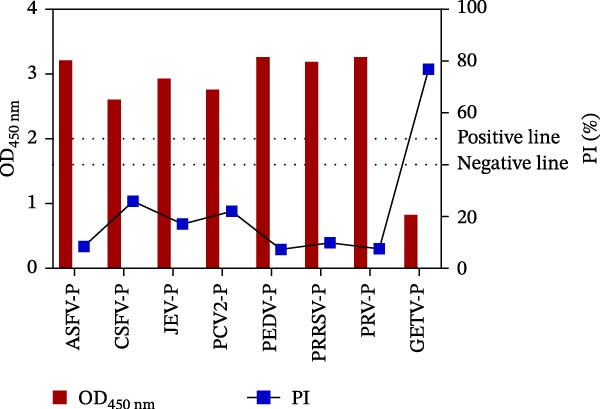
Analytical specificity and sensitivity of the Blocking ELISA. Percent inhibition (PI) values of sera positive for different porcine viruses were evaluated. Only GETV‐positive sera showed PI values exceeding the cutoff threshold, indicating high analytical specificity and sensitivity of the assay.

To determine the analytical specificity of the blocking ELISA, serum samples from pigs with seven other common swine viruses (ASFV, CSFV, JEV, PCV2, PEDV, PRV, and PRRSV) were tested. GETV‐positive serum was included as a positive control. The results showed that only the GETV‐positive sample yielded a high PI, while no cross‐reactivity was observed with sera from pigs infected with the other viruses (Figure 7). These findings confirm that the blocking ELISA specifically detects GETV antibodies, a key requirement for reliable diagnosis and differentiation of GETV infections in swine populations.

### 3.6. Repeatability of the Blocking ELISA

To evaluate the repeatability of the blocking ELISA, 10 GETV antibody–positive and eight negative serum samples were tested under intra‐ and interassay conditions. The intra‐assay CV for PI ranged from 0.395% to 9.767% when the same samples were tested in triplicate on the same plate (Table 3). Interassay CV ranged from 0.0716% to 7.749% across three different batches of precoated plates (Table 4). These data indicate that the blocking ELISA has good repeatability, as CV values below 10% are generally considered acceptable in serological assays [21].

**Table 3:** Intra‐assay reproducibility of the GETV blocking ELISA.

Serum samples	Group 1		Group 2		Group 3		*x̄*	SD	CV (%)
	PI (%)	Results	PI (%)	Results	PI (%)	Results			
1	81.807	+	82.636	+	82.236	+	82.226	0.338	0.411
2	86.867	+	88.695	+	88.199	+	87.920	0.771	0.877
3	86.024	+	84.697	+	85.466	+	85.396	0.544	0.637
4	83.373	+	83.948	+	82.981	+	83.434	0.397	0.476
5	94.759	+	94.503	+	93.851	+	94.371	0.382	0.405
6	94.398	+	93.379	+	93.416	+	93.731	0.472	0.503
7	95.000	+	94.691	+	94.099	+	94.597	0.374	0.395
8	70.783	+	69.644	+	68.199	+	69.542	1.058	1.521
9	22.651	−	27.358	−	26.894	−	25.634	2.118	8.263
10	15.745	−	16.677	−	13.168	−	15.196	1.484	9.767
11	26.929	−	21.487	−	23.354	−	23.923	2.258	9.439
12	23.690	−	19.675	−	19.565	−	20.977	1.919	9.149
13	19.511	−	18.176	−	16.538	−	18.075	1.216	6.725
14	13.373	−	13.191	−	12.236	−	12.934	0.499	3.857
15	4.208	−	3.534	−	3.791	−	3.845	0.278	7.222
16	8.158	−	8.620	−	8.199	−	8.325	0.209	2.507
17	8.156	−	8.245	−	8.920	−	8.441	0.341	4.043
18	4.475	−	4.997	−	4.907	−	4.793	0.228	4.751

**Table 4:** Interassay reproducibility of the GETV blocking ELISA.

Serum samples	Group 1		Group 2		Group 3		x̄	SD	CV (%)
	PI (%)	Results	PI (%)	Results	PI (%)	Results			
1	83.455	+	81.306	+	82.725	+	82.495	0.892	1.082
2	87.044	+	88.002	+	89.537	+	88.194	1.027	1.164
3	86.540	+	87.723	+	88.229	+	87.497	0.708	0.809
4	80.651	+	83.147	+	86.649	+	83.482	2.460	2.947
5	94.223	+	95.368	+	95.313	+	94.968	0.527	0.555
6	94.840	+	95.592	+	95.913	+	95.448	0.449	0.471
7	95.457	+	95.480	+	96.131	+	95.689	0.312	0.326
8	67.078	+	66.964	+	74.768	+	69.604	3.652	5.247
9	15.415	−	15.844	−	14.659	−	15.306	0.490	3.199
10	23.101	−	22.727	−	25.241	−	23.690	1.108	4.675
11	25.463	−	24.049	−	22.345	−	23.952	1.275	5.321
12	23.972	−	20.137	−	22.509	−	22.206	1.580	0.071
13	16.067	−	16.817	−	14.303	−	15.729	1.054	6.699
14	14.764	−	15.433	−	13.556	−	14.584	0.777	5.326
15	4.539	−	4.908	−	4.414	−	4.620	0.210	4.535
16	8.451	−	9.919	−	9.866	−	9.412	0.680	7.222
17	9.150	−	7.645	−	8.012	−	8.269	0.641	7.749
18	7.585	−	6.453	−	6.718	−	6.919	0.483	6.987

### 3.7. Clinical Application of the Blocking ELISA

**Figure 8:**
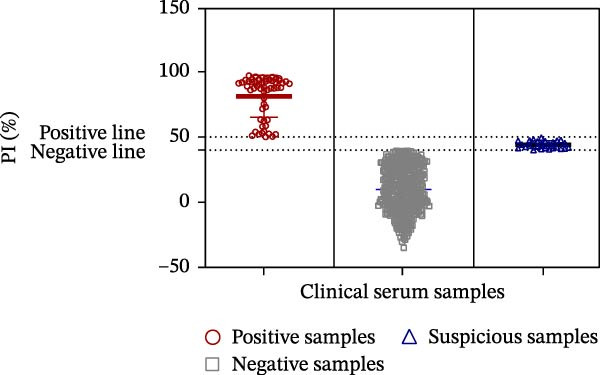
Serological detection of anti‐GETV antibodies in clinical pig serum samples using the developed blocking ELISA. Among the 462 samples tested, 56 (12.121%) were positive, 26 (5.628%) were classified as suspicious, and 380 (82.251%) were negative based on the established percent inhibition (PI) criteria.

To evaluate the practical applicability of the developed GETV E2 blocking ELISA, a total of 462 porcine serum samples were collected between 2022 and the summer of 2023. The samples were obtained from the Animal Disease Diagnostic Center at Huazhong Agricultural University. Based on the PI thresholds established in this study, the assay was successfully applied for the serological detection of GETV antibodies in field samples (Figure 8), demonstrating its potential utility for epidemiological surveillance and routine diagnostic use in swine populations.

## 4. Discussion

In this study, we developed a novel blocking ELISA for the detection of GETV antibodies in swine, targeting the highly conserved E2 protein. The assay demonstrated high specificity and sensitivity, and screening of 462 clinical swine serum samples revealed a seroprevalence of 12.121% in China, confirming ongoing field circulation of GETV [1]. This work addresses a critical gap in veterinary diagnostics by providing a standardized, reproducible tool for large‐scale serological surveillance, thereby supporting epidemic preparedness and informing control strategies in the swine industry.

The choice of E2 as the target antigen is particularly advantageous [22]. E2 is a highly conserved structural protein of GETV, playing a key role in viral attachment and entry and eliciting a strong neutralizing antibody response [23]. Its conserved and immunodominant nature makes it an ideal target for serological assays, ensuring broad detection across different viral strains while minimizing cross‐reactivity. Compared with previous studies that used capsid or partially characterized E2 fragments, the use of full‐length, eukaryotically expressed E2 in our assay preserves native protein folding, enhancing the generation of conformation‐sensitive mAbs [24]. Notably, mAb 1D reacted in IFA but not in Western blot, strongly suggesting the recognition of a conformational epitope disrupted during SDS‒PAGE, highlighting the importance of antigen conformation for assay design.

The observed seroprevalence aligns with reports from other regions in Asia, supporting widespread GETV circulation, while methodological refinements improve reproducibility and applicability across diverse herd settings. Limitations include potential sampling bias and the need for field validation. Stratified sampling across provinces and herd types, along with prospective multicenter evaluation, would further enhance the generalizability and reliability of the assay.

Future research could expand surveillance to other susceptible livestock, integrate molecular characterization of circulating GETV strains, and develop predictive models linking seroprevalence with environmental and vector‐related factors. Longitudinal studies and field‐based validation of the blocking ELISA would strengthen its utility in outbreak management and vaccine evaluation, providing a comprehensive framework for GETV control and surveillance.

## 5. Conclusions

In conclusion, this study makes a meaningful contribution to GETV diagnostics by establishing a sensitive, specific, and reproducible blocking ELISA. This method offers a rapid and scalable approach for the detection of GETV antibodies, providing a valuable tool for epidemiological surveillance and outbreak response. Nonetheless, one limitation of the current work is the lack of epitope mapping to define the antigenic determinants of the E2 protein. Future studies focusing on identifying linear B‐cell epitopes and evaluating the neutralizing capacity of mAbs will be essential to deepen our understanding of GETV immunology. Such efforts will not only enhance diagnostic capabilities but also inform the rational design of targeted vaccines, ultimately advancing both diagnostic and preventive strategies for managing this emerging arboviral threat.

## Funding

This work was supported by the earmarked fund for the National Key R&D Program of China (Grant 2025YFC3507700), the CARS (Grant CARS‐35), and the “Yingzi Tech & Huazhong Agricultural University Intelligent Research Institute of Food Health” (Grant IRIFH202209).

## Ethics Statement

This study was approved by the Scientific Ethics Committee of Huazhong Agricultural University (HZAU) and was performed according to animal ethics guidelines and approved protocol (HZAUMO‐2024‐0137).

## Conflicts of Interest

The authors declare no conflicts of interest.

## Data Availability

The data that support the findings of this study are available from the corresponding author upon reasonable request.
